# Silver Finance—Asset Management of Elderly People in the Modern Era

**DOI:** 10.1093/geroni/igaa057.1427

**Published:** 2020-12-16

**Authors:** Frances Yang, Ka Hin, Edward Cheung, Vivian Lou

**Affiliations:** 1 the University of Hong Kong, Hong Kong, China; 2 The University of Hong Kong, Hong Kong, China

## Abstract

This study aims to explore the elderly people’s knowledge, attitude, and understandings toward financial management, and how they behave in using IT-supported financial services. Mixed methodologies, including quantitative street survey and qualitative focus group interviews, are used. The result shows an active involvement with investment market of the mature segments despite claiming safe and stable returns as the paramount principle of asset management. They also have a substantial knowledge on some of the technological-enabled financial services, particularly the younger groups (age 50-59). Cybersecurity is their major concern and barrier on whether to use them or not. Besides, their value and behaviors of buying mature adult financial products should be understood within a context of traditional family culture value. Based on the above analysis, the elderly can be categorized into three major types in using IT-supported financial products and services, including proactive users, passive followers and hard opposition. On the one hand, this research breaks up the outdated stereotype of technology use among elderly people; on the other hand, aged people are facing the challenges of cognitive and physical decline led by ageing. As such, it is essential to conduct specific financial literacy intervention. This project brings new insights into the financial education which is appropriate to the elderly.

